# Storage Stability of Chilled and Frozen Starch Gels as Affected by Blended Starch Formulation, Sucrose Syrup, and Coconut Milk

**DOI:** 10.1155/2022/9454229

**Published:** 2022-03-01

**Authors:** Aumaporn Arlai, Kanitha Tananuwong

**Affiliations:** ^1^Department of Food Science and Technology, Faculty of Science and Technology, Nakhon Pathom Rajabhat University, Nakhon Pathom 73000, Thailand; ^2^Department of Food Technology, Faculty of Science, Chulalongkorn University, Bangkok 10330, Thailand

## Abstract

Effects of starch formulation, highly concentrated sucrose solution, and coconut milk on the stability of starch gels kept under chilled and frozen conditions were determined. Gels containing rice starch (RS), tapioca starch (TS) (RS:TS of 1 : 0.85), and hydroxypropyl distarch phosphate (HDP, 0-50% of total starch) were prepared from 15% starch suspension using water, 45°Brix sucrose syrup or coconut milk as liquid media. After aging at 4°C for 21 days, starch gels had higher hardness and chewiness, with lower cohesiveness and springiness (*p* ≤ 0.05). Water-based gels containing HDP had less extent of texture hardening, lower degree of crystallinity, and more homogeneous microstructure during 4°C aging. However, for the starch gels in sucrose syrup or coconut milk, HDP induced greater gel hardening, higher degree of crystallinity, and denser gel microstructure during chilled storage. This could be due to the crystallization of sucrose or lipid/amylose-lipid complexes. Nevertheless, HDP enhanced freeze-thaw stability of the gels, regardless of the liquid media used (*p* ≤ 0.05). According to the consumer test of the model desserts subjected to a single freeze-thaw cycle, the sample containing 50% HDP gel in sucrose syrup or 25% HDP gel in coconut milk gained the highest hedonic score of texture and overall acceptance (*p* ≤ 0.05).

## 1. Introduction

Rice-tapioca starch blend has widely been used in ASEAN traditional dessert recipes. In comparison with rice starch gel, the gels containing rice-tapioca starch blends had a lower degree of starch retrogradation, slower changes in the gel texture during chilled and frozen storage, and better freeze-thaw stability [1, 2]. Nevertheless, changes in textural parameters of the starch-based desserts during storage were not only induced by starch retrogradation but also influenced by sugars and lipids in the formulation. Sucrose and coconut milk are the general ingredients incorporated in ASEAN desserts. Concentrated sucrose syrup and coconut milk were shown to improve freeze-thaw stability of the starch gels. However, those ingredients accelerated an increase in hardness of the starch gels during chilled temperature storage, presumably due to sugar crystallization and formation of amylose-lipid complex crystallites [2]. Therefore, modification of the blended starch recipe was required to mitigate the effects of those ingredients and thus retard the changes in gel texture during low temperature storage.

Stabilized or substituted starches have generally been used as a texture modifier in food products. These modified starches were shown to reduce hardness with enhancing the elasticity of the starch-based foods. The main mechanism underlying such texture modification was decreasing degree of retrogradation via the bulky substituted groups, including an esterified acetyl group and etherified hydroxypropyl group [3]. In this study, hydroxypropyl distarch phosphate (HDP), a dual modified tapioca starch containing hydroxypropyl group, was selected as a representative of the starch with low retrogradation ability. HDP also had high stability to shear force, thermal processing, and low pH caused by the cross-linked phosphate groups. However, the function of the hydroxypropyl group in HDP was mainly focused in this work. Efficiency of hydroxypropylated starch on retarding the change in texture of the starch gels during long-term cold storage, as well as improving their freeze-thaw stability, was recently reviewed elsewhere [4]. However, those water-based starch gels were mostly prepared from a single type of starch. A study on the application of hydroxypropylated starch, as well as HDP, in more complicated gel systems, including gels containing ≥2 types of starches and/or prepared with concentrated sugar solution or emulsified liquid medium, was still limited. The aim of this research was to evaluate the influence of blended starch formulation and the type of aqueous media used for the gel preparation on the stability of starch gels during chilled and frozen storage. Reduced retrogradation ability of hydroxypropylated starch might help counterbalance the effects of sugar and lipid recrystallization and alleviate the changes in texture-related quality of the blended starch gels under cold storage. Mechanisms underlying those changes were also proposed based on X-ray diffractograms and microstructure of the gels. Consumer preference of the model dessert containing the blended starch gels in different aqueous media was also determined. The overall results could provide a guideline for improving the quality of the Asian dessert containing rice/tapioca starch gel, sucrose syrup, and coconut milk.

## 2. Materials and Methods

### 2.1. Material

Rice starch (33.75% amylose) and tapioca starch (31.4% amylose) were obtained from Cho Heng Rice Vermicelli Co., Ltd. (Nakhon Pathom, Thailand) and Thai Tham Factory (Chonbury, Thailand), respectively. Hydroxypropyl distarch phosphate (HDP) derived from tapioca starch was supplied by Siam modified starch (Pathumthani, Thailand). Food grade sucrose (Mitr Phol Sugar Co. Ltd., Supanburi, Thailand) and coconut milk (Thai Agri Food Co., Ltd., Nakhon Pathom, Thailand) were purchased from local market.

### 2.2. Starch Gel Preparation

Composition of the starch blends is shown in Table 1. To the prepared starch suspension (15% *w*/*w* of starch, dry basis), the blended starch sample was mixed with potable water, 45°Brix sucrose solution, or coconut milk and continuously stirred at 250 rpm for 5 min. The suspension was then heated to 80°C, held for 20 min under continuous stirring at 200 rpm, and subsequently poured into a rectangular mold (4 × 5 × 1 cm^3^) or 150 ml centrifuge tubes for gel formation. The gel samples in the rectangular molds were kept at 4°C for 3 hours to obtain the freshly prepared gels or stored at 4*°*C for 21 days before further analyses. The starch gels in the centrifuge tubes were kept frozen (-18°C) before evaluating freeze-thaw stability and microstructure.

### 2.3. Determination of Starch Gel Properties during Chilled Storage

Hardness of the freshly prepared gels and the aged starch gels was determined by Texture analyzer (TA.XT plus, UK), using the method of Arlai and Tananuwong [2]. Briefly, the starch gel in rectangular cuboid shape (4 × 5 × 1 cm^3^) was placed at room temperature until its temperature reached 25 ± 3°C. Texture profile analysis was performed using P/100 cylindrical probe, 40% strain of compression level, and 1 mm/s of test speed. Crystallinity pattern found in the aged starch gels was evaluated from the X-ray diffractogram. Samples were prepared with the similar method explained in Arlai and Tananuwong [2]. Bruker D8 Discover X-ray diffractometer (Bruker AXS, Germany) was used in the analysis. The degree of crystallinity was calculated as the area percentage of the crystalline region based on the total peak area (amorphous and crystalline region) using Origin® 2019 software (OriginLab Corporation, MA, USA).

### 2.4. Freeze-Thaw Stability of the Starch Gels

Freeze-thaw stability of the gel samples was determined by the method listed in Arlai and Tananuwong [2]. Single freeze-thaw cycle required 7 days of frozen storage. For each cycle, the centrifugation technique was used to evaluate the degree of syneresis. The warmed samples were centrifuged at 15000 × g for 15 min. The degree of syneresis was calculated from the following equation. To eliminate the effects of solutes and emulsified lipids in the liquid phase, the degree of syneresis was calculated based on the total weight of water in the gel.

(1) $$\begin{array}{c} \text{Degree of syneresis }\left(\%\right)=\frac{\text{Liquid separated after centrifugation }\left(\text{ml}\right)}{\text{Total weight of water in the gel samples }\left(\text{g}\right)}\times 100. \end{array}$$

### 2.5. Microstructure of the Chilled and Frozen Starch Gels

Microstructure of aged starch gels, kept chilled or frozen, was determined by a scanning electron microscope (SEM) (Tescan mira3, Czech Republic), under 50x magnification and 15 kV accelerated voltage. Sample preparation was previously explained [2].

### 2.6. Sensory Evaluation of the Model Dessert

#### 2.6.1. Model Dessert Preparation

**Figure 1:**
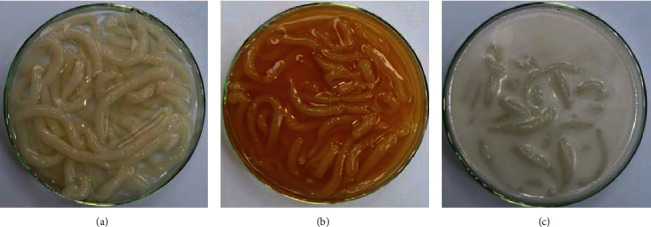
Appearance of the model desserts prepared in water (a), 45°Bx sucrose solution (b), and coconut milk (c).

Model dessert based on the traditional Thai dessert (Plakrim-Kaitow) was prepared using the starch blend, having the starch composition as listed in Table 1. For starch dough formation, the starch blend was mixed with 0.1% Ca(OH)_2_ solution at the weight ratio of 0.8 : 1. The dough was steamed for 5 min, kneaded for 10–15 min, and rested at room temperature for 30 min. The dough was extruded through the perforated plate (0.5 cm pore diameter) into boiling water, hot sucrose solution (45°Brix), or hot coconut milk. The noodle-like thick starch strands were heated in the selected liquid medium for 5 min. The temperature of water, sucrose solution, or coconut milk was maintained at 100 ± 2°C during the heating step. The weight ratio of the liquid medium to starch strands was 3 : 1. The gelatinized starch strands were then removed from the heating medium, packed with additional water, 45°Brix sucrose solution or coconut milk, corresponding to the liquid medium used in the heating step. Appearance of the final products is shown in Figure 1. The samples were stored at -18°C for 7 days, thawed, and used for sensory analysis (equivalent to 1 freeze-thaw cycle).

#### 2.6.2. Sensory Evaluation

The sensory evaluation for consumer acceptance testing was performed by 30 untrained panelists, 20–55 years old, composed of 5 men and 25 women. All panelists were familiar with this kind of traditional Thai dessert. The 9-point hedonic scale questionnaire (1 = dislike extremely, 9 = like extremely) was used to evaluate the consumer preference of the freshly prepared samples and freeze-thawed samples. Test attributes were appearance, color, flavor, texture (softness), and overall acceptance of the dessert. Temperature of the samples used for sensory evaluation was 35 ± 2°C.

### 2.7. Statistical Analysis

A completely randomized design was applied to experiments related to the physical properties of the starch gels. Randomized complete block design was used for the sensory test. The analysis of variance was performed. Difference among means was evaluated by Duncan's new multiple range test at the confidence level of 95%. SPSS software version 23 (IBM, USA) was used to conduct all statistical analyses.

## 3. Results and Discussion

### 3.1. Properties and Microstructure of the Chilled Starch Gels

Textural parameters of the aged starch gels in different aqueous media are shown in Table 2. At similar storage time (0 or 21 days, 4°C) and the aqueous medium used, starch gels containing HDP had lower hardness and chewiness than that of the control (0% HDP). The samples containing 50% HDP had the lowest gel hardness and chewiness (*p* ≤ 0.05). However, the cohesiveness of both freshly prepared and aged gels was not affected by HDP and the type of aqueous medium used (*p* > 0.05). Springiness of the freshly prepared gels was not influenced by these two factors (*p* > 0.05). Nevertheless, the springiness of the aged samples prepared with concentrated sucrose solution was the lowest, regardless of the HDP addition (*p* ≤ 0.05). Softer texture of the blended starch gels containing HDP could be due to the lower degree of starch retrogradation. Application of HDP neither increased brittleness nor elasticity (as seen from similar cohesiveness and springiness values, respectively) of the gels. In addition, long-term aging at chilled temperature induced an increase in hardness and chewiness, with a decrease in cohesiveness and springiness of the gels. The degree of change in hardness and chewiness of the gels largely depended on the level of HDP used.

For the gels prepared from starch-water suspension, starch retrogradation was proposed as the major phenomenon causing an increase in gel hardness and chewiness during storage at 4°C. It was clearly seen that HDP effectively retarded gel hardening during chilled storage. Lower percentage of the increase in those textural parameters was evidenced in the formula containing greater proportion of HDP (*p* ≤ 0.05). This could result from the poor retrogradation ability of HDP. The steric hindrance of the hydroxypropyl functional group was shown to lessen the interaction between amylose/amylopectin chains; thus, the starch retrogradation was retarded [5, 6]. However, the greater level of HDP did not efficiently prevent the loss of springiness and cohesiveness of the water-based gels, as evidenced by the nearly similar extent of those changes among the samples (7.3%–8.4% reduction in springiness and 0–1.2% reduction in cohesiveness). As for the samples prepared in heavy syrup or coconut milk, the larger degree of gel hardening during chilled storage was evidenced in the samples containing HDP. Starch gels containing higher proportion of HDP had a greater percentage of the increase in hardness and chewiness after 21 days storage at 4°C (*p* ≤ 0.05). This could be due to the different mechanisms, apart from starch retrogradation, that induced the hardening process of aged starch gels in the presence of sugar and emulsified fats [2].

**Figure 2:**
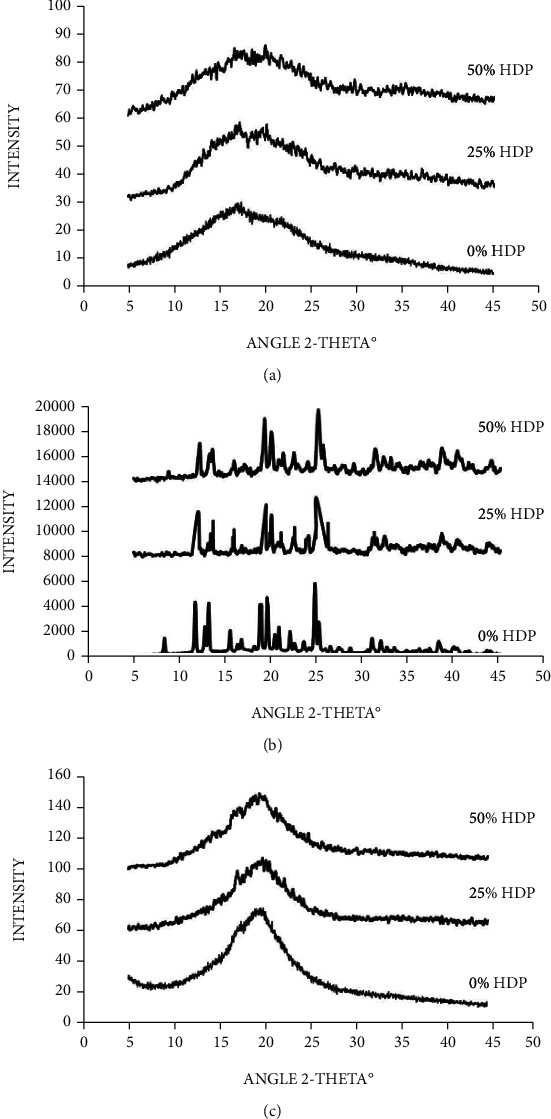
X-ray diffractograms of the starch gels in water (a), 45°Bx sucrose solution (b), or coconut milk (c) after storage at 4oC for 21 days. The gels were prepared with the starch blends containing different levels of HDP substitution as shown in Table 1.

According to our previous study, multiple phenomena could occur during chilled storage of rice-tapioca starch gels prepared with concentrated sucrose syrup or coconut milk, including starch retrogradation, sucrose recrystallization, and amylose-lipid complex formation [2]. Based on the X-ray diffractogram shown in Figure 2, those occurrences were also evidenced in the blended starch gels containing HDP. In the water-based gels (Figure 2(a)), small peaks at 2*θ* of 17° and 20°, representing B-type crystallinity of the retrograded starch and amylose-lipid complex V-type crystallinity [7, 8], were observed. However, strong signals of sucrose crystallinity, having 2*θ* of 11.8°, 12.8°, 18.9°, 19.7°, 20.9°, 24.8°, 25.2°, 31.2°, and 32.1° [9], were found in the starch gels prepared with concentrated sucrose syrup (Figure 2(b)). According to the gels containing coconut milk, the peaks at 2*θ* of 17° and 20° were also found (Figure 2(c)). However, the stronger signal was evidenced at 2*θ* around 19°-20°. This could also arise from fat crystallization in the gel samples. Szydłowska-Czerniak et al. [10] reported the wide angle X-ray diffractogram of fat mixtures comprising rapeseed oil, soybean oil, palm oil, and coconut oil, isothermally crystallized at 22°C for 60 min. The peaks at 2*θ* of 18.97°–19.28° were assigned to the *β*-form of the fat crystalline structure. In the case of coconut milk, thermograms obtained from differential scanning calorimetry (DSC) showed the exothermic peaks of fat crystallization, having peak temperature of 5°C and 2°C, upon cooling of the homogenized coconut milk from 30°C to -15°C [11]. Ariyaprakai and Tananuwong [12] also reported the DSC exothermic peaks of fat crystallization in emulsion systems containing coconut oil and specific types of surfactants. A peak having onset temperature of 8.2°C–8.8°C and peak temperature of 6.1°C–7.7°C was shown in the DSC thermogram. Based on these DSC studies, fat crystallization could occur in the starch gels prepared with coconut milk and stored at 4°C for 21 days. Certain polymorphic forms of the fat crystalline structures could also contribute to the peak at 2*θ* of 19°-20° in the X-ray diffractogram. Therefore, apart from starch retrogradation, sucrose and fat crystallization could play an important role on the hardening of starch gels incorporated with sucrose and coconut milk, respectively, during chilled storage.

**Figure 3:**
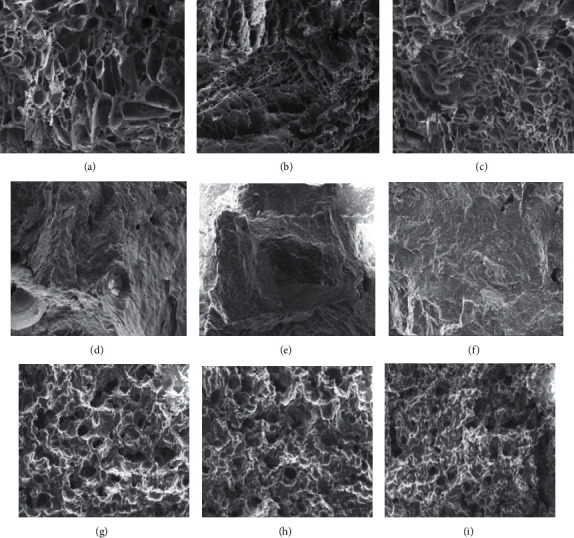
Microstructure of the aged starch gels containing 0%HDP (a, d, g), 25%HDP (b, e, h), and 50%HDP (c, f, i). The gels were prepared in water (a–c), 45°Bx sucrose solution (d–f), or coconut milk (g–i) and kept at 4°C for 21 days.

The microstructure of the aged starch gels in various aqueous media is shown in Figure 3. Water-based starch gels depicted a porous structure, representing the gel network with entrapped water. As HDP content increased, the gel with relatively smaller pores and more uniform structure was obtained (Figures 3(b) and 3(c)). HDP could enhance starch-water interactions and thus decreased starch retrogradation during storage, resulting in gels with a denser and more uniform structure. Dun et al. [7] reported the changes in the microstructure of rice starch gels after storage at 4°C for 7 days. According to the SEM images of rice starch gel, the freshly prepared gels had a dense porous structure. However, the network of aged starch gels became loosen, having larger pores in the matrix, representing the repulsion of water from the gel network due to starch retrogradation. Therefore, the looser gel network could imply to the greater degree of starch retrogradation, as evidenced in this study. The SEM results thus agreed well with the textural parameters of the starch gels in water (Table 2) and the degree of crystallinity of the aged gels (Table 3). HDP efficiently retarded the retrogradation of blended starch gel during chilled storage, resulting in softened gels with denser and more uniform structure. Lower degree of crystallinity found in the gels with greater proportion of HDP (*p* ≤ 0.05) could be due to less extent of starch retrogradation occurred during chilled storage. Nevertheless, HDP seemed to provide smaller effects on the microstructure of the starch gels prepared with concentrated sucrose solution or coconut milk.

The extremely dense structure of the starch gels in sucrose syrup is shown in Figures 3(d)–3(f). It was interesting to note that this gel system contained only 47% moisture, compared to 85% moisture in the water-based starch gels. Therefore, the dense gel structure could result from the limited amount of water and sucrose recrystallization during storage. The latter phenomena was evidenced from X-ray diffractogram (Figure 2(b)). We hypothesized that sucrose crystallization was the major mechanism of gel hardening during aging at 4°C. In the gel system with HDP, a greater degree of starch-water interaction was obtained, resulting in less water available for hydration of sucrose. Higher degree of supersaturation could further induce sucrose crystallization. This presumption was supported by the degree of crystallinity shown in Table 3. The gels with higher HDP content had greater degree of crystallinity (*p* ≤ 0.05), indicating that more sucrose crystals were formed. These gel samples thus had a larger increase in gel hardness and chewiness (Table 2). Nevertheless, the extent of the reduction in springiness and cohesiveness was relatively similar among the aged samples containing 0–50% HDP, similarly to the results from the water-based system. It was also interesting to note that, among the three aqueous media used, the greatest reduction in springiness and cohesiveness was evidenced in the sucrose syrup-based gels (*p* ≤ 0.05). Blended starch gels containing recrystallized sucrose became more brittle and less elastic.

As for the starch gels prepared with coconut milk, a denser gel network with less porous structure was obtained (Figures 3(g)–3(i)), in comparison with the structure of the water-based gels (Figures 3(a)–3(c)). Coconut milk is an oil-in-water emulsion which also contains proteins, sugars, and minerals. Therefore, the available water in the gel system was reduced to 67%. This could induce the formation of a denser gel matrix. Similar to the microstructure of the starch gels in water, the gels with higher proportion of HDP tended to have a more uniform structure with smaller pores distributed in the matrix. This might result from the retrogradation retarding effects of HDP as well as oil-in-water emulsion characteristic of coconut milk. Impact of oil-in-water emulsion containing 5% soybean oil on delaying the retrogradation of rice starch was previously reported [7]. According to the SEM images, the rice starch gel prepared with that emulsion system and aged at 4°C for 7 days had more uniform and compact structure in comparison with the gel sample in water. However, a greater increase in hardness and chewiness, with a larger decrease in cohesiveness (Table 2) and higher degree of crystallinity (Table 3), was found in the aged coconut milk-based gels containing higher levels of HDP. X-ray diffraction pattern could indicate the existence of amylose-lipid complex (V-type polymorph) and fat crystallization in the aged gel samples. During starch gel preparation, the starch suspension was heated to 80°C for 20 min. Such thermal processing was shown to induce the destabilization of coconut milk emulsion [13]. The breakage of some oil droplets could enhance the amylose-lipid formation. Despite the crosslinking via the esterified phosphate groups, the bulky hydroxypropyl substituted groups might enhance the swelling and solubility of the HDP, which could increase the accessibility of amylose molecules during gelatinization. Greater extent of amylose-lipid complex might then be formed in the systems containing higher levels of HDP. Complexation between amylose and lipids was reported to occur during gelatinization and cooling of the starch suspension [14–16]. Additional formation of the amylose-lipid complex and/or its crystalline perfection was less likely to occur during storage at 4°C. On the other hand, time-dependent fat crystallization at chilled temperature occurred, including triglycerides in the coalesced and aggregated lipid droplets embedded in the gel matrix. Therefore, we speculated that coconut milk-based gels containing HDP could have a greater degree of amylose-lipid complex formation and/or fat crystallization. These occurrences eventually resulted in hardened and more brittle gels, with a higher degree of crystallinity, during chilled temperature storage.

The overall results from this section indicated that the addition of HDP in rice-tapioca starch gels might not effectively retard textural changes during prolonged storage at chilled temperature. The type of aqueous media used to prepare gel samples played an important role in such changes. Starch retrogradation might not be the only phenomenon that induced these quality changes. In the system containing high solute concentration, particularly sugars used in traditional dessert formula, crystallization of solutes during aging could be the main mechanism underlying the changes in gel texture. For the complex gel system containing emulsion, both fat crystallization and amylose-lipid complex formation could highly impact the textural quality of the aged starch gels [2].

### 3.2. Freeze-Thaw Stability and Microstructure of the Frozen Starch Gels

Syneresis of the different gel systems after freezing and thawing is presented in Table 4. At similar freeze-thaw cycle (except the 1st cycle) and blended starch composition, the water-based gel systems had the greatest degree of syneresis, followed by the gels in coconut milk and sucrose syrup, respectively. Water binding ability of sucrose and modified ice recrystallization due to surface active agents was proposed for the lower degree of syneresis in the starch gels incorporated with sucrose syrup and coconut milk, respectively [2]. Unlike the aged starch gels kept at chilled temperature, HDP provided beneficial effects in every frozen gel systems. As HDP content increased, the degree of syneresis gradually decreased (*p* ≤ 0.05). Starch modification via hydroxypropylation and crosslinking was shown to reduce the syneresis in taro starch [6], rice starch [17], and sago starch [18]. This could indicate that the syneresis was mainly driven by starch retrogradation. The hydrophilic hydroxypropyl group could effectively enhance starch-water interactions, thus lessening the degree of syneresis of freeze-thawed gels [5].

**Figure 4:**
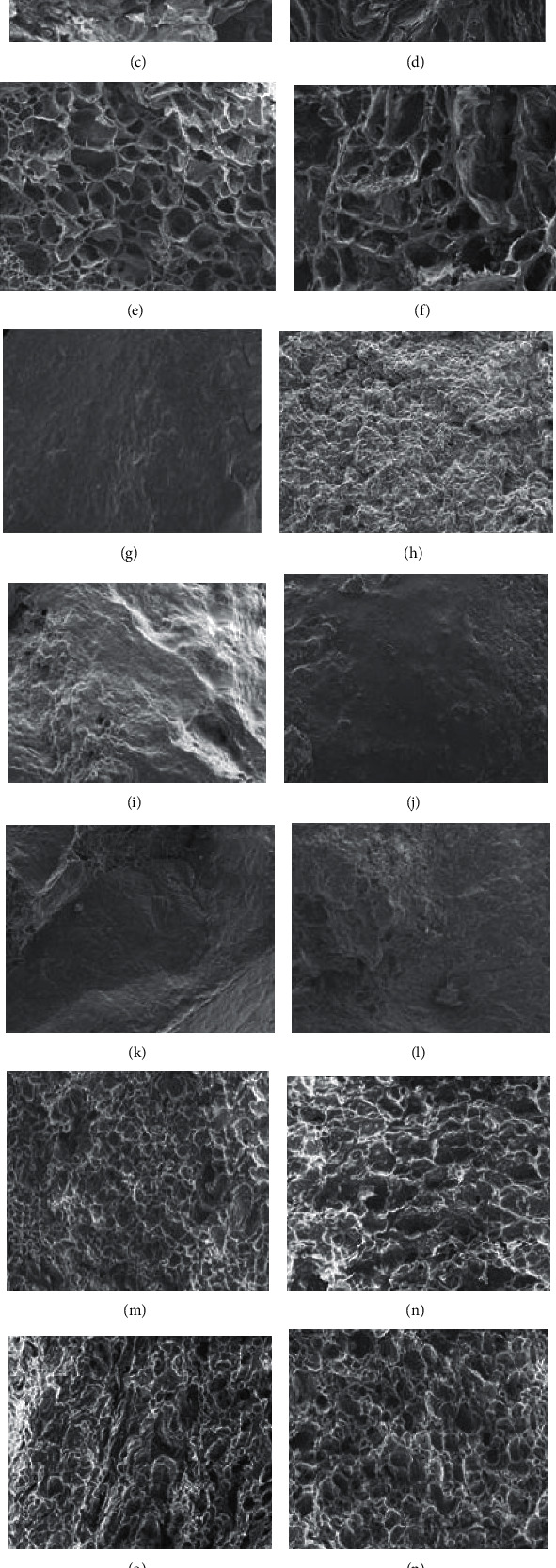
Microstructure of the frozen starch gels containing 0%HDP (a, d, g, j, m, p), 25%HDP (b, e, h, k, n, q), and 50%HDP (c, f, i, l, o, r). The gels were prepared in water (a–f), 45°Bx sucrose solution (g–l), or coconut milk (m–r) and subjected to 1 (a–c, g–i, m–o) or 5 (d–f, j–l, p–r) freeze-thaw cycles.

Microstructure of the freeze-thaw starch gels is depicted in Figure 4. For the starch gel in water, pore enlargement after the 5^th^ freeze-thaw cycle was seen in all samples (Figures 4(a)–4(c), in comparison with Figures 4(d)–4(f)), corresponding to the increasing degree of syneresis as freeze-thaw progressed (Table 4). This microstructural change was most obvious in the 0%HDP sample. The pattern of the changes in the microstructure of the coconut milk-based gels containing various levels of HDP and subjected to different numbers of freeze-thaw cycles was similar to that found in the water-based gels (Figures 4(m)–4(r)). However, the extent of such changes in the coconut milk-based gels was smaller, which was in an agreement with lower degree of syneresis (*p* ≤ 0.05, Table 4). For the gels in sucrose syrup which had the lowest degree of syneresis, the gel microstructure was least affected by HDP and number of freeze-thaw cycles (Figures 4(g)–4(l)).

### 3.3. Sensory Evaluation of the Model Dessert

HDP was clearly shown to reduce hardness and chewiness but minimally influenced springiness and cohesiveness of the starch gels in different aqueous media (Table 2) and improve their freeze-thaw stability (Table 3) (*p* ≤ 0.05). Hence, it was interesting to evaluate if HDP addition could help improve consumer preference of the desserts containing those starch gels. In the model dessert, preparation of starch gels was modified to minimize the effects of sucrose and emulsified lipids on the gel texture. Dough of the blended starch was prepared and subsequently cooked in water, sucrose syrup, or coconut milk. According to this approach, sucrose and lipids could mainly migrate into the exterior part of the gels and might not thoroughly distribute within the starch gels. Textural modified effects of sucrose and lipids could thus mainly occur at the outer part, while the inner core could maintain the characteristics of blended starch gels. This approach could help mitigate the undesirable effects of sucrose and lipid crystallization on the starch gel texture during storage.

Results from the consumer acceptance test of the fresh and frozen model dessert comprising starch gels with HDP are shown in Table 5. For the freshly prepared desserts using a specific type of aqueous media, the hedonic scores of appearance, color, and flavor of the desserts containing 0–50% of HDP in starch gels were relatively similar. This could indicate that HDP provided negligible effects on those attributes. As for the preference in gel texture of the unaged desserts, HDP slightly influenced the hedonic scores of softness of the water-based and coconut milk-based starch gels. However, for the samples prepared in sucrose syrup, the hedonic score of softness of the gels containing HDP was higher than that of the control (*p* ≤ 0.05). An increase in the overall acceptability scores of these HDP added samples (*p* ≤ 0.05) could mainly result from a greater preference in the gel texture. Softness enhancing ability of HDP, without losing the elasticity of the gels, was thus desirable in the unaged desserts prepared with sucrose syrup.

Effects of HDP on enhancing consumer preference of the model desserts were evidenced in the frozen samples (Table 5). For any aqueous media used, while HDP slightly affected the preference in appearance, color, and flavor, it clearly increased the hedonic scores of softness and overall acceptability of the desserts subjected to 1 freeze-thaw cycle (*p* ≤ 0.05). This could be due to the lower degree of starch retrogradation in the samples containing HDP, which could retard the changes in gel texture during freezing and thawing. Interestingly, for the dessert samples containing sucrose syrup, the gels containing 50% HDP gained the highest hedonic scores of softness and overall acceptability (*p* ≤ 0.05). However, the desserts with 25% and 50% HDP gels in coconut milks obtained similar hedonic scores of softness and overall acceptability (*p* > 0.05). Therefore, a higher level of HDP might be required to enhance the consumer acceptability of the dessert samples prepared with sucrose syrup.

## 4. Conclusions

HDP has long been used as a texture modifier in starch-based products, particularly those requiring long-term cold storage. For freshly prepared gels in water, sucrose syrup, and coconut milk, the formula with a greater level of HDP had lower hardness and chewiness, with the slight difference in springiness and cohesiveness. When considering the stability of normal starch gels, especially those containing low concentration of solutes and lipids, retrogradation was the major mechanism inducing the changes in texture-related quality of the gels during cold storage. Therefore, HDP could be applied successfully to retard those quality changes. However, in complex gel systems containing high concentration of solutes and/or emulsified lipids, stability of the gels could be affected by various phenomena. According to the samples aged under chilled temperature, this study indicated that the crystallization of sucrose and lipid/amylose-lipid complexes greatly influenced the texture of rice-tapioca starch gels incorporated with sucrose syrup and coconut milk, respectively. In those circumstances, HDP could not effectively retard the gel hardening during storage. However, HDP could enhance freeze-thaw stability of all samples, regardless of the aqueous media used in the gel preparation. The ability of HDP to improve the sensory quality of the rice-tapioca starch gels in different aqueous media was also determined in the model dessert. HDP was shown to enhance consumer acceptance of the freshly prepared starch gels in sucrose syrup. Additional benefits of HDP were reflected in the frozen dessert prepared with sucrose syrup and coconut milk. Addition of 25% HDP in the gel formula was sufficient to maximize the overall acceptability of the freeze-thaw samples containing coconut milk. However, for the dessert prepared with sucrose syrup, 50% of HDP was required to achieve the highest overall acceptability. Overall results from this study could expand the viewpoint for the application of HDP in complex starch-based food systems, including the ASEAN traditional desserts.

## Figures and Tables

**Table 1:** Composition of starch blends used in this study.

Hydroxypropylated distarch phosphate substitution level (%)	Weight ratio of rice starch	Weight ratio of tapioca starch	Weight ratio of hydroxypropylated distarch phosphate
0	54.0	46.0	0.0
25	40.5	34.5	25.0
50	27.0	23.0	50.0

**Table 2:** Textural parameters of the freshly prepared starch gels and the gels stored at 4°C for 21 days.

Starch gels	Textural parameters of the starch gels							
	Freshly prepared gels				Aged at 4°C for 21 days			
	Hardness (*N*)	Springiness^ns^	Cohesiveness^ns^	Chewiness (*N*)	Hardness (*N*)	Springiness	Cohesiveness^ns^	Chewiness (*N*)
Water								
0% HDP	15.11^d^ ± 0.49	0.85 ± 0.02	0.84 ± 0.01	10.79^c^ ± 0.19	24.63^e^ ± 1.90 (+63.00^g^ ± 0.80)	0.78^a^ ± 0.03 (−8.24^c^ ± 0.29)	0.84 ± 0.01 (0)	16.14^d^ ± 0.09 (+49.58^d^ ± 1.17)
25% HDP	14.32^de^ ± 1.01	0.83 ± 0.05	0.81 ± 0.02	9.63^d^ ± 0.26	19.85^f^ ± 1.29 (+38.61^h^ ± 1.06)	0.76^a^ ± 0.04 (−8.43^c^ ± 0.14)	0.80 ± 0.03 (−1.23^c^ ± 0.12)	12.07^f^ ± 0.54 (+25.36^g^ ± 1.04)
50% HDP	13.34^f^ ± 0.95	0.82 ± 0.03	0.82 ± 0.05	8.97^e^ ± 0.13	17.84^g^ ± 0.69 (+33.73^i^ ± 0.84)	0.76^a^ ± 0.05 (−7.32^d^ ± 0.18)	0.81 ± 0.04 (−1.22^c^ ± 0.26)	10.98^g^ ± 0.08 (+22.44^h^ ± 0.88)
45°Bx sucrose solution								
0% HDP	23.67^a^ ± 0.48	0.83 ± 0.02	0.83 ± 0.06	16.31^a^ ± 0.65	39.89^b^ ± 4.17 (+68.52^f^ ± 1.55)	0.67^b^ ± 0.05 (−19.28^a^ ± 0.35)	0.81 ± 0.05 (−2.41^b^ ± 0.18)	21.65^b^ ± 0.87 (+32.76^f^ ± 1.08)
25% HDP	19.95^b^ ± 0.54	0.81 ± 0.02	0.82 ± 0.02	13.25^b^ ± 0.17	34.58^bc^ ± 3.46 (+73.33^e^ ± 1.40)	0.66^b^ ± 0.05 (−18.52^a^ ± 0.54)	0.80 ± 0.12 (−2.44^b^ ± 0.16)	18.26^c^ ± 0.62 (+37.79^e^ ± 1.03)
50% HDP	11.13^h^ ± 0.47	0.82 ± 0.03	0.81 ± 0.05	7.39^f^ ± 0.08	29.37^d^ ± 3.66 (+163.88^a^ ± 1.89)	0.69^b^ ± 0.02 (−15.85^b^ ± 0.17)	0.79 ± 0.10 (−2.47^b^ ± 0.11)	16.01^e^ ± 0.95 (+116.56^a^ ± 3.72)
Coconut milk								
0% HDP	23.99^a^ ± 0.39	0.83 ± 0.04	0.83 ± 0.01	16.53^a^ ± 0.10	46.10^a^ ± 1.76 (+92.16^d^ ± 0.95)	0.77^a^ ± 0.08 (−7.23^d^ ± 0.36)	0.83 ± 0.02 (0)	29.46^a^ ± 1.11 (+78.27^c^ ± 1.19)
25% HDP	16.99^e^ ± 1.70	0.82 ± 0.05	0.83 ± 0.03	11.56^c^ ± 0.45	33.01^c^ ± 4.17 (+94.29^c^ ± 1.23)	0.76^a^ ± 0.05 (−7.32^d^ ± 0.20)	0.82 ± 0.06 (−1.20^c^ ± 0.28)	20.57^b^ ± 0.98 (+77.90^c^ ± 0.92)
50% HDP	10.67^g^ ± 0.23	0.84 ± 0.05	0.84 ± 0.02	7.53^f^ ± 0.21	24.65^e^ ± 2.01 (+131.02^b^ ± 1.72)	0.78^a^ ± 0.06 (−7.14^d^ ± 0.57)	0.81 ± 0.05 (−3.57^a^ ± 0.25)	15.57^e^ ± 0.86 (+106.86^a^ ± 4.84)

^a,b,…^Mean values with different letters in the same column were significantly different (*p* ≤ 0.05). For the aged starch gels, data in the brackets represented the percentage of changes based on that of the freshly prepared gels. The gels were prepared from starch blends as shown in Table 1. HDP: hydroxypropylated distarch phosphate.

**Table 3:** Degree of crystallinity of the starch gels stored at 4°C for 21 days.

Aqueous media used for gel preparation	Degree of crystallinity of the gels containing different level of HDP		
	0%	25%	50%
Water	5.86^g^ ± 0.05	5.59^h^ ± 0.12	4.85^i^ ± 0.13
45°Bx sucrose solution	64.77^c^ ± 0.24	68.64^b^ ± 0.55	73.37^a^ ± 0.43
Coconut milk	5.43^f^ ± 0.32	5.73^e^ ± 0.16	6.41^d^ ± 0.14

^a,b…^For all 9 treatments, mean values with different letters were significantly different (*p* ≤ 0.05). The gels were prepared from starch blends as shown in Table 1. HDP: hydroxypropylated distarch phosphate.

**Table 4:** Degree of syneresis of the frozen starch gels after freezing and thawing up to 5 cycles.

Starch gels	Degree of syneresis (%)				
	1 cycle	2 cycles	3 cycles	4 cycles	5 cycles
Water					
0% HDP	14.94^a^ ± 1.52	34.07^a^ ± 3.58	38.43^a^ ± 4.55	42.97^a^ ± 3.48	43.49^a^ ± 0.87
25% HDP	0	29.92^b^ ± 4.68	36.37^b^ ± 3.50	40.24^b^ ± 0.85	41.36^b^ ± 2.90
50% HDP	0	22.91^c^ ± 4.29	34.15^c^ ± 1.20	38.05^c^ ± 3.02	40.67^b^ ± 2.81
45°Bx sucrose solution					
0% HDP	0	0	0	0	1.95^f^ ± 1.54
25% HDP	0	0	0	0	1.16^f^ ± 2.01
50% HDP	0	0	0	0	0.38^h^ ± 0.67
Coconut milk					
0% HDP	0	22.15^c^ ± 5.35	29.44^d^ ± 2.68	33.55^d^ ± 0.65	35.32^c^ ± 2.53
25% HDP	0	17.51^d^ ± 2.67	24.57^e^ ± 5.81	27.23^e^ ± 0.79	30.32^d^ ± 1.96
50% HDP	0	13.40^e^ ± 2.72	18.09^f^ ± 6.83	19.68^f^ ± 2.91	22.28^e^ ± 3.09

^a,b…^Mean values with different letters in the same column were significantly different (*p* ≤ 0.05). The gels were prepared from starch blends as shown in Table 1. HDP: hydroxypropylated distarch phosphate.

**Table 5:** Hedonic scores of the freshly prepared and frozen model dessert using different formulations of starch blends and aqueous media.

Dessert samples	Freshly prepared dessert					Frozen dessert subjected to 1 freeze-thaw cycle				
	Appearance	Color	Flavor	Softness	Overall acceptability	Appearance	Color	Flavor	Softness	Overall acceptability
Water										
0%HDP	8.30^c^ ± 0.11	8.50^b^ ± 0.09	8.00^b^ ± 0.08	8.00^c^ ± 0.18	8.00^c^ ± 0.08	7.80^c^ ± 0.12	8.00^a^ ± 0.13	8.00^b^ ± 0.13	7.70^d^ ± 0.12	7.70^c^ ± 0.12
25%HDP	8.38^c^ ± 0.13	8.57^b^ ± 0.09	8.10^b^ ± 0.08	8.30^b^ ± 0.11	8.20^bc^ ± 0.09	8.38^b^ ± 0.10	8.20^a^ ± 0.21	8.20^b^ ± 0.11	8.00^c^ ± 0.08	8.00^b^ ± 0.13
50%HDP	8.30^c^ ± 0.09	8.20^c^ ± 0.22	8.00^b^ ± 0.10	7.80^c^ ± 0.20	8.00^c^ ± 0.08	8.30^b^ ± 0.11	8.20^a^ ± 0.21	8.00^b^ ± 0.13	8.30^b^ ± 0.11	8.00^b^ ± 0.13
45°Bx sucrose solution										
0%HDP	8.60^b^ ± 0.15	8.89^a^ ± 0.22	8.50^a^ ± 0.27	7.00^c^ ± 0.08	7.50^d^ ± 0.21	8.60^ab^ ± 0.18	8.00^a^ ± 0.13	8.50^a^ ± 0.23	7.00^e^ ± 0.21	7.00^d^ ± 0.21
25%HDP	8.50^bc^ ± 0.12	8.78^a^ ± 0.14	8.45^a^ ± 0.22	8.50^ab^ ± 0.14	8.20^bc^ ± 0.09	8.50^ab^ ± 0.12	8.10^a^ ± 0.13	8.45^ab^ ± 0.21	8.00^c^ ± 0.08	8.00^b^ ± 0.13
50%HDP	8.55^bc^ ± 0.17	8.75^a^ ± 0.14	8.50^a^ ± 0.30	8.50^ab^ ± 0.14	8.70^a^ ± 0.13	8.55^ab^ ± 0.17	8.20^a^ ± 0.21	8.50^a^ ± 0.23	8.50^ab^ ± 0.12	8.50^a^ ± 0.12
Coconut milk										
0%HDP	8.70^ab^ ± 0.11	8.89^a^ ± 0.21	8.60^a^ ± 0.26	8.70^a^ ± 0.10	8.50^bc^ ± 0.21	8.70^a^ ± 0.20	8.00^a^ ± 0.13	8.50^a^ ± 0.23	8.00^c^ ± 0.08	8.00^b^ ± 0.13
25%HDP	8.80^a^ ± 0.07	8.78^a^ ± 0.12	8.65^a^ ± 0.23	8.80^a^ ± 0.09	8.70^a^ ± 0.13	8.72^a^ ± 0.18	8.00^a^ ± 0.13	8.60^a^ ± 0.23	8.60^a^ ± 0.18	8.72^a^ ± 0.18
50%HDP	8.85^a^ ± 0.10	8.75^a^ ± 0.15	8.60^a^ ± 0.25	8.85^a^ ± 0.12	8.90^a^ ± 0.18	8.69^a^ ± 0.18	8.15^a^ ± 0.21	8.60^a^ ± 0.23	8.80^a^ ± 0.12	8.61^a^ ± 0.18

^a,b…^Mean values with different letters in the same column were significantly different (*p* ≤ 0.05). Hedonic scores were reported in the 9-point scale; 1 = dislike extremely, 9 = like extremely. The gels were prepared from starch blends as shown in Table 1. HDP: hydroxypropylated distarch phosphate.

## Data Availability

Data used to support the findings are included in this article. Additional information is available from the corresponding author upon request.

## References

[1] Seetapan N., Limparyoon N., Gamonpilas C., Methacanon P., Fuongfuchat A. (2015). Effect of cryogenic freezing on textural properties and microstructure of rice flour/tapioca starch blend gel. *Journal of Food Engineering*.

[2] Arlai A., Tananuwong K. (2021). Quality of chilled and frozen starch gels as affected by starch type, highly concentrated sucrose and coconut milk. *LWT*.

[3] Masina N., Choonara Y. E., Kumar P. (2017). A review of the chemical modification techniques of starch. *Carbohydrate Polymers*.

[4] Fu Z., Zhang L., Ren M.-H., BeMiller J. N. (2019). Developments in hydroxypropylation of starch: a review. *Starch*.

[5] Zhu F. (2015). Composition, structure, physicochemical properties, and modifications of cassava starch. *Carbohydrate Polymers*.

[6] Hazarika B. J., Sit N. (2016). Effect of dual modification with hydroxypropylation and cross-linking on physicochemical properties of taro starch. *Carbohydrate Polymers*.

[7] Dun H., Liang H., Zhan F. (2020). Influence of O/W emulsion on gelatinization and retrogradation properties of rice starch. *Food Hydrocolloids*.

[8] Niu H., Zhang M., Xia X., Liu Q., Kong B. (2018). Effect of porcine plasma protein hydrolysates on long-term retrogradation of corn starch. *Food Chemistry*.

[9] Chinachoti P., Steinberg M. P. (1986). Crystallinity of sucrose by X-ray diffraction as influenced by absorption versus desorption, waxy maize starch content, and water activity. *Journal of Food Science*.

[10] Szydłowska-Czerniak A., Karlovits G., Lach M., Szłyk E. (2005). X-ray diffraction and differential scanning calorimetry studies of *β*′ -> *β* transitions in fat mixtures. *Food Chemistry*.

[11] Tangsuphoom N., Coupland J. N. (2009). Effect of thermal treatments on the properties of coconut milk emulsions prepared with surface-active stabilizers. *Food Hydrocolloids*.

[12] Ariyaprakai S., Tananuwong K. (2015). Freeze-thaw stability of edible oil-in-water emulsions stabilized by sucrose esters and Tweens. *Journal of Food Engineering*.

[13] Raghavendra S. N., Raghavarao K. S. M. S. (2010). Effect of different treatments for the destabilization of coconut milk emulsion. *Journal of Food Engineering*.

[14] Le Bail P., Bizot H., Ollivon M., Keller G., Bourgaux C., Buléon A. (1999). Monitoring the crystallization of amylose–lipid complexes during maize starch melting by synchrotron x-ray diffraction. *Biopolymers*.

[15] Kwaśniewska-Karolak I., Nebesny E., Rosicka-Kaczmarek J. (2008). Characterization of amylose-lipid complexes derived from different wheat varieties and their susceptibility to enzymatic hydrolysis. *Food Science and Technology International*.

[16] Wang S., Chao C., Cai J., Niu B., Copeland L., Wang S. (2020). Starch–lipid and starch–lipid–protein complexes: a comprehensive review. *Comprehensive Reviews in Food Science and Food Safety*.

[17] Deetae P., Shobsngob S., Varanyanond W., Chinachoti P., Naivikul O., Varavinit S. (2008). Preparation, pasting properties and freeze-thaw stability of dual modified crosslink-phosphorylated rice starch. *Carbohydrate Polymers*.

[18] Wattanachant S., Muhammad K., Mat Hashim D., Rahman R. A. (2003). Effect of crosslinking reagents and hydroxypropylation levels on dual-modified sago starch properties. *Food Chemistry*.

